# Morphological and functional phenotyping of skeletal muscle and bone in the zQ175 knock-in mouse model of Huntington's disease

**DOI:** 10.1177/18796397251387208

**Published:** 2025-10-28

**Authors:** Behnaz Nateghi, Mohamed Lala Bouali, Zineb Bouredji, Anteneh Argaw, Soher Nagi Jayash, Colin Farquharson, Jérôme Frenette, Sébastien S Hébert

**Affiliations:** 1 Axe Neurosciences, 4440 Centre de Recherche du CHU de Québec-Université Laval, CHUL, Québec, Canada; 2 Faculté de Médecine, Département de Psychiatrie et de Neurosciences, Université Laval, Québec, Canada; 3 The Roslin Institute and Royal (Dick) School of Veterinary Studies, 3124 University of Edinburgh, Easter Bush, Midlothian, UK; 4 École des Sciences de la Réadaptation, Faculté de Médecine, Université Laval, Québec City, Canada

**Keywords:** Huntington's disease, zQ175, muscle atrophy, bone microarchitecture, musculoskeletal dysfunction, knock-in mousemodel

## Abstract

**Background**: Huntington's disease (HD) is a progressive neurodegenerative disorder primarily affecting the central nervous system (CNS). However, emerging evidence suggests that peripheral tissues, including skeletal muscle and bone, also undergo pathological changes contributing to disease burden. **Objective**: To characterize musculoskeletal impairments in the zQ175 knock-in (KI) mouse model of HD, through integrated behavioral, biomechanical, and imaging analyses. **Methods**: Motor function was assessed using grip strength, rotarod, and open field testing. *Ex vivo* contractility of the extensor digitorum longus (EDL) and Soleus (Sol) muscles was measured. Muscle fiber cross-sectional area (CSA) was quantified using semi-automated segmentation. Bone microarchitecture was analyzed using high-resolution micro-computed tomography (μCT). **Results**: Six-month-old homozygous zQ175 mice exhibited significantly reduced muscle strength and impaired contractile properties in both the EDL and Soleus muscles compared to wild-type (WT) controls. µCT analysis revealed decreased trabecular bone volume and alterations in bone structure. **Conclusions**: These findings provide a comprehensive musculoskeletal phenotyping of zQ175 mice, revealing early-onset muscle atrophy and skeletal fragility. Our study highlights the importance of targeting peripheral manifestations in HD and establishes zQ175 KI mice as a valuable additional model for investigating systemic disease mechanisms.

## Introduction

Huntington's disease (HD) is a rare neurodegenerative disorder that primarily affects the central nervous system (CNS). It is caused by an abnormal expansion of the CAG trinucleotide repeat in the *Huntingtin* (HTT) gene, located on the short arm of chromosome 4 (4p16.3). This expansion leads to an elongated polyglutamine (polyQ) tract in the HTT protein. The widely accepted threshold for disease onset is 36 or more CAG repeats, with fully penetrant clinical manifestations typically occurring from 40 upwards.^1^ Higher repeats of CAG are associated with earlier onset, faster disease progression, and greater severity. The expanded polyglutamine tract in exon 1 of HTT promotes misfolding and aggregation of the mutant HTT protein (mHTT), forming insoluble aggregates that accumulate throughout disease progression.^2^ These aggregates have been identified not only in the brain but also in peripheral tissues, including skeletal muscle.^3,4^ Thus, while traditionally most research has focused on the role of HTT in the brain, accumulating studies underscore the importance of understanding the molecular mechanisms underlying skeletal muscle deterioration in HD.^5–7^ In particular, targeting peripheral tissues such as skeletal muscle could represent a promising strategy to improve the quality of life for HD patients.^8,9^ Interestingly, disease onset could be delayed and lifespan extended by improving muscle function in mice.^10,11^

One common non-neurological feature of HD is progressive weight loss, which may appear early in the disease course. Individuals with higher body mass index (BMI) at early stages tend to experience slower disease progression, indicating that weight loss might serve as a prognostic marker. This weight loss reflects deeper changes in body composition, including significant reductions in muscle mass and signs of abnormal bone mineral density (BMD), particularly in the later stages of the disease, although detailed longitudinal studies are still lacking.^12^

In transgenic R6/2 mice overexpressing the human mHTT exon 1 fragment with expanded CAG repeats, BMD loss occurred before significant weight loss, pointing to an active, HD-intrinsic process rather than a secondary consequence of muscle deterioration.^13^ Notably, muscle atrophy and loss of BMD often occur in parallel across various physiological and pathological conditions.^14,^^15^ Thus, early detection of musculoskeletal deterioration in HD is clinically relevant and may offer opportunities for timely interventions to prevent disability. Current research efforts are intensely focused on accurately modeling these peripheral pathologies.

It has been suggested that the limb and trunk skeletal muscles of HD patients are affected during disease progression, causing significant postural instability.^16,17^ Other investigations in transgenic HD mouse models, including R6/2 and N171-82Q, have reported skeletal muscle atrophy linked to mitochondrial dysfunction and altered energy metabolism.^18^ Although behavioural and motor studies are well documented in HD models (e.g., R6/2, YAC128, BACHD, and HdhQ111),^19^ concomitant functional or anatomical studies of the musculoskeletal system remain unexplored.

The zQ175 knock-in (KI) mouse model closely mimics the onset and progressive development of HD by preserving the endogenous structure and regulatory context of the mutant HTT gene. In this study, we used zQ175 homozygous mice to perform a comprehensive analysis of behavioral, functional, and anatomical alterations in skeletal muscle and bone at an early to mid-symptomatic age (6 months). We first quantified motor and neuromuscular performance using open field, grip strength, and rotarod assays. We then assessed muscle contractility, morphology, and bone microarchitecture *via* contractile force measurements and micro-computed tomography (µCT). Our results reveal marked impairments in muscle strength and locomotor activity, consistent with earlier findings.^20,21^ These functional losses were associated with intrinsic abnormalities in skeletal muscle and bone tissues. Collectively, these findings underscore the utility of zQ175 and potentially other KI mouse models in studying peripheral manifestations in HD.

## Research design and methods

### Study design

This study followed a descriptive experimental design to evaluate muscle atrophy and associated functional deficits in HD. Both motor behavior and structural changes in muscle and bone were assessed in homozygous zQ175 KI HD mice and sex and age-matched wild-type (WT) littermate controls. A combination of behavioral assays, *ex vivo* muscle physiology, histological analysis, and imaging techniques was employed to capture a comprehensive picture of disease-associated changes.

### Animals

**Figure 1.**
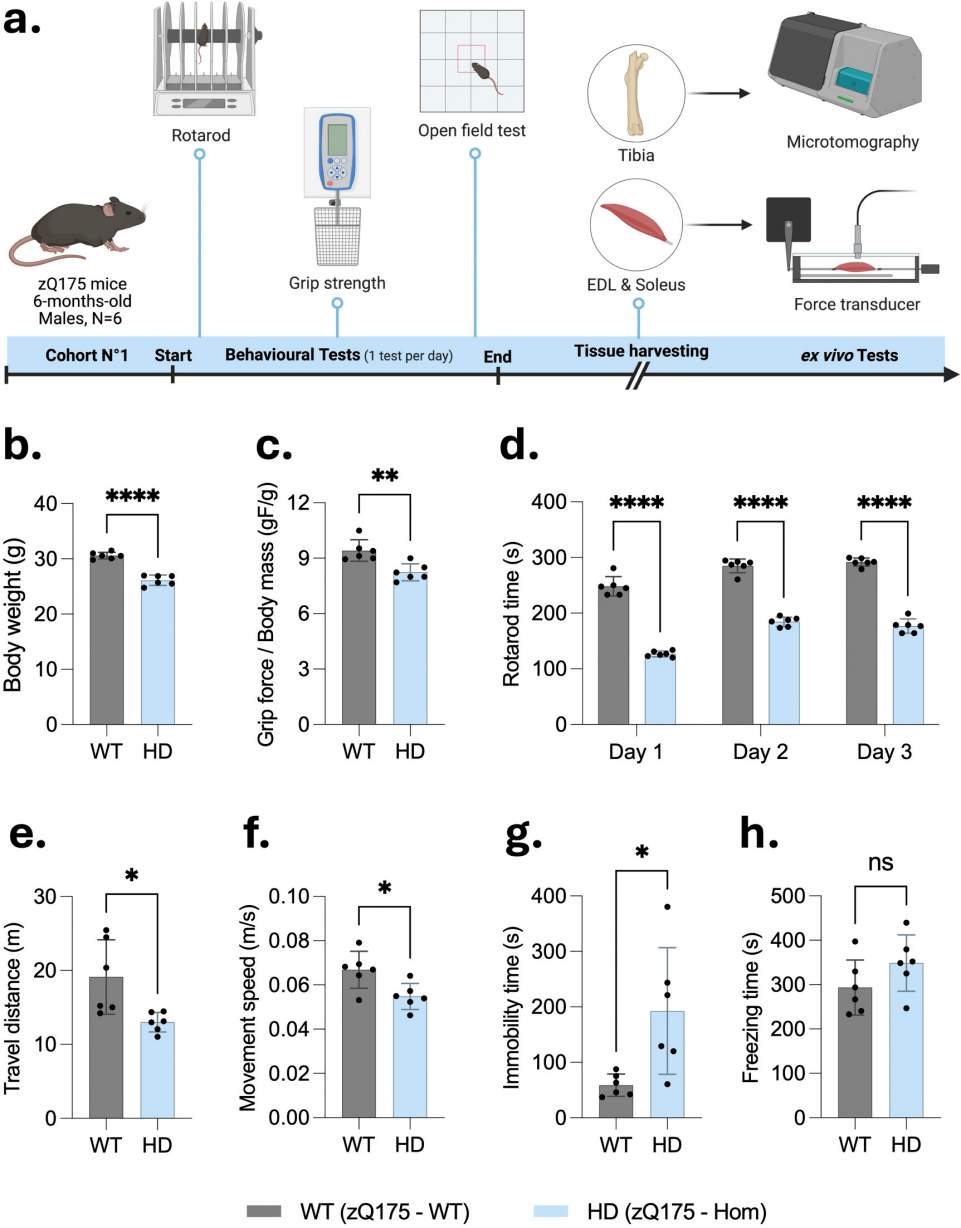
Behavioral deficits and reduced physical performance in 6-month-old homozygous zQ175 mice. (a) Schematic representation of the experimental timeline for the first cohort of mice (n = 6 per group, all males), which underwent a series of behavioral tests - rotarod, grip strength, and open field - prior to tissue harvesting and *ex vivo* analyses (Figures 1, 2, 3, 5 and 6). Created with BioRender.com. (b–c) The four-limb grip force performances were significantly reduced in homozygous zQ175 (HD) mice compared to wild-type (WT) controls when normalized to body weight. (d) Rotarod performance revealed a significantly lower latency to fall across three consecutive days in HD mice. (e–f) In the open field test, HD mice displayed significantly reduced total travel distance and average movement speed. (g) Immobility time was increased in HD mice, while (h) freezing time did not differ significantly between genotypes. All data are presented as mean ± SD. Statistical analyses were performed using unpaired two-tailed Student's t-test or parametric multiple unpaired t-test corrected for multiple comparisons using the Holm-Šídák method for Rotarod data. *p < 0.05, **p < 0.01, ***p < 0.001, ****p < 0.0001; ns: not significant.

The experimental procedures involving mice were carried out in agreement with the guidelines provided by the Canadian Council on Animal Care and were approved by the Animal Care Committee of Université Laval (CPAUL-3, approval number: CHU-21-902). Male and female homozygous zQ175 mice with WT littermate controls, all aged 6 months, were used in this study. Two independent cohorts were included: Cohort 1 (n = 6, all males) was used for the majority of experiments (see Figure 1(a)), including behavioral tests, *ex vivo* muscle function analyses, and bone assessments; Cohort 2 (n = 5; 3 males and 2 females per group) was used exclusively for muscle morphometric analyses (see Figure 4(a)). Due to the absence of viable homozygous females in our breeding pairs, potential sex-related differences were not investigated in this study. The mice were derived from a colony maintained within our animal facilities and were originally obtained from The Jackson Laboratory. Homozygous zQ175 mice and WT littermates were generated by crossing heterozygous zQ175 knock-in mice carrying the human *HTT* exon 1 sequence with a ∼190 CAG repeats (JAX, Strain #027410) on a C57BL/6J background (JAX, Strain #000664). Mice were housed in a controlled environment at 22°C temperature, with a 12:12 h light/dark cycle, with *ad libitum* access to food and water. Genotyping was performed *via* PCR using genomic DNA extracted from ear biopsies then confirmed once again from tail after sacrifice.

### Behavioral testing

Behavioral assessments were conducted during the light phase in a dedicated testing room. Mice were allowed to acclimatize for 30 min before testing, and all equipment was cleaned with 70% ethanol between subjects.

### Grip strength

Grip test was conducted as indicated before.^19^ Briefly, the four-limb muscular force of 6-month-old mice was measured using a digital force meter (Columbus Instruments, Columbus, OH, USA). Mice were held by the tail and allowed to grasp a horizontal metal grid. The peak force exerted was recorded across three trials, with a minimum 1-min interval between measurements. Maximum grip strength was normalized to body weight.

### Open field

Locomotor activity was assessed in a 50 × 50 cm open-field arena using the Any-maze tracking system (Stoelting Co., Wood Dale, IL, USA) for 16 h (overnight). Mice were placed in the center of the arena and allowed to explore freely after a 10-min habituation period. Total distance traveled, immobility time (periods when the mouse ceases active movement but may still make small postural adjustments), movement speed, and freezing time (complete lack of movement except for respiration) were recorded.

### Rotarod

Motor coordination was evaluated using a rotarod apparatus (Panlab/Harvard). Mice underwent one training trial (5 min at 4 RPM) per day for three days. Testing consisted of three 5-min accelerating trials (0–40 RPM over 300 s) per day with 30-min inter-trial intervals. The latency to fall was recorded, with a maximum cutoff of 300 s.

### Skeletal muscle contractile properties

Mice were injected subcutaneously with buprenorphine (0.1 mg/kg) to reduce pain and were anesthetized intra-peritoneally with pentobarbital sodium (50 mg/kg). The extensor digitorum longus (EDL) and Soleus (Sol) muscles, representing one of the slowest and one of the fastest skeletal muscles, respectively, were isolated. Contractile properties were measured using a 305B-LR dual-mode muscle arm system controlled by a dynamic muscle data acquisition and analysis system (Aurora Scientific Inc., Aurora, ON, Canada). The muscles were incubated at 25°C in Krebs-Ringer physiological solution (pH 7.4) supplemented with 95% O2 and 5% CO2 and the muscles were positioned between two parallel platinum electrodes for stimulation, while the tendons were secured to the force transducer and a fixed hook to record contractile properties. A single twitch contraction was obtained, and tetanic contractions were elicited at frequencies of 10 to 150 Hz by 1-min rest periods to generate force-frequency curves. Maximum specific tetanic tension (sP0 in N/cm2) values were obtained by normalizing the absolute force (P0) with the cross-sectional area (CSA) using the following equation: sP0 = P0/CSA. CSA was determined by dividing the muscle mass by the product of the optimum fiber length (Lf) corresponding to the result of multiplying optical length (L0) by the fiber length ratio (0.44 for EDL muscles and 0.71 for Sol muscles) and the muscle density (1.06 mg/mm3).^22^ Briefly, following a 10-min equilibration period, optimal muscle length (L_0_) was determined by delivering a single 1 Hz twitch every 30 s and adjusting the resting muscle length until maximal twitch force was achieved. Optimal voltage was established by applying supramaximal stimulation with a bi-phasic stimulator (Aurora Scientific, 701C) through two parallel platinum electrodes (∼9 mm apart) flanking the muscle in the bath. Stimulation parameters were then applied consistently across all groups to ensure full fiber recruitment and avoid suboptimal activation. The muscle contractility results were analyzed using Dynamic Muscle Data Analysis software (Aurora Scientific Inc.). Muscles were weighed after drying to determine their mass.

### Myofiber immunostaining and image acquisition

A dedicated cohort of sex and age-matched 6-month-old mice was used for muscle atrophy assessment *via* myofiber cross-sectional area (CSA) measurement (n = 5 per genotype, 3 males and 2 females per group). EDL and Soleus muscles from the right limb were carefully dissected from anesthetized mice and were incubated in Ringer's solution at 4°C until embedded in Optimal Cutting Temperature (OCT) compound (FisherScientific, #23-730-571) and rapidly frozen using liquid nitrogen-cooled isopentane and stored at −80°C until further analysis. Cryosections (10 µm thickness) were prepared using a cryostat at −20°C, mounted onto positively charged glass slides (FisherScientific, #22-037-246), and stored at −20°C until staining. Muscle cross-sections were immunostained with an anti-laminin antibody to clearly delineate individual myofibers. Briefly, slides were fixed with cold acetone, blocked with PBS supplemented with 1% horse serum and 2% bovine serum albumin, and incubated overnight at 4°C with primary anti-laminin (Sigma-Aldrich, #L9393, 1:1000) antibody diluted in the same buffer. The sections were washed briefly with PBS, incubated with Alexa-Fluor-488-conjugated secondary antibody (Invitrogen, #A-11034, 1:500) for 1 h at room temperature and washed three times for 15 min with PBS. Muscle sections were finally counter-stained for nucleus using 4′,6-diamidino-2-phenylindole (DAPI)-containing Fluoromount-G (FisherScientific, #5018788) and left to dry overnight at room temperature. Images were acquired using a Zeiss Axio Imager.Z2 upright microscope equipped with an LSM 800 confocal system and an EC Plan-Neofluar 10×/0.30 M27 air objective, using the stitching function in ZEN 2 software to acquire high-resolution images covering the entire surface of each muscle section.

### Segmentation and quantitative image analysis of myofiber cross-sectional area (CSA)

Accurate segmentation of individual myofibers were conducted using Fiji software (version 1.54f) integrated with the Cellpose and LabelToROI plugins as previously described.^23^ In brief, single-channel laminin fluorescence images were segmented with Cellpose using a python script on GoogleColab.^23^ Resulting labeled images were processed with LabelToROI to generate Fiji regions of interests (ROIs). These ROIs were eroded with a fixed 2 pixels to accurately delineate the boundaries of myofibers based on visual inspection. Each image was then thoroughly inspected manually to ensure correct and accurate selection of myofibers. Acquired measurements were then adjusted for pixel size and normalized myofiber cross-sectional areas expressed in squared micrometer (μm^2^). For statistical analysis, the mean CSA of all myofibers within a single cross-section was calculated for each mouse and used as one independent data point (n = 5 per group), in accordance with current best practices to ensure independence of observations. These same cross-sections were also used to generate CSA frequency distribution histograms, in which the CSA of every individual myofiber from each section contributed to the bin counts.

### Cortical and trabecular bone analysis by microcomputed tomography

The cortical geometry and trabecular architecture of tibia were determined using a microcomputed tomography (μCT) (Skyscan 1172 X-ray microtomography, Bruker, Kontich, Belgium). In brief, high-resolution scans with an isotropic voxel size of 5 μm were acquired (60 kV, 167 μA and 0.5 mm filter, 0.6° rotation angle). The slices were then reconstructed using the NRecon 1.7.3.0 program (Bruker, Kontich, Belgium). CTAn software 1.15.4.0 (Skyscan) was used to visualize and determine bone histomorphometric parameters from the reconstructed image sets. The selected trabecular (Tb) region of interest (ROI) in the proximal tibial metaphysis began from the bottom of the growth plate, excluding the cortical shell and extended 5% of the entire tibial length. A total of 250 slices beneath this 5% were selected to exclude the primary spongiosa. Within the diaphysis, 100 slices were selected for the analysis of cortical bone, with the ROI starting at distances of 50% from the proximal end of the tibia. Cortical BMD was calculated using hydroxyapatite rod pair phantoms of a known density (0.25 g/cm^3^ and 0.75 g/cm^3^) which were scanned using the same settings used for cortical image accrual.

### Statistical analysis

All graphics and statistical analyses were performed using GraphPad Prism 10 Software (Graph Pad Software, Inc., La Jolla, CA, USA). All data are expressed as means ± SD. Parametric tests were applied after confirming normality (see Figure legends). Group comparisons were conducted using unpaired two-tailed Student's t-test, force-frequency curves were analyzed using two-way ANOVA with Bonferroni post hoc correction, and rotarod tests we analyzed using multiple unpaired t-tests with Holm-Šídák correction for multiple comparisons. Statistical significance was defined as follows: p < 0.05 (*), p < 0.01 (**), p < 0.001 (***), p < 0.0001 (****).

## Results

### Mid-aged homozygous zQ175 mice display deficits in locomotor activity and muscle strength

As depicted in Figure 1(a), 6-month-old homozygous zQ175 mice and littermate WT controls underwent behavioral testing (see Methods). At this age, homozygous zQ175 mice displayed a significantly lower body mass compared to WT controls (Figure 1(b)). Muscle strength was measured using a grip strength test. The four-limb grip force was normalized to body mass (gF/g) for both genotypes. HD mice demonstrated significantly reduced normalized grip strength compared to WT counterparts (Figure 1(c)).

Motor coordination and balance were assessed using the rotarod test across three consecutive days. HD mice showed a consistently reduced latency to fall compared to WT mice, indicating significant motor deficits (Figure 1(d)). To evaluate voluntary locomotor activity, mice were monitored overnight in an open field using a video tracking system. HD mice exhibited markedly reduced activity levels compared to age-matched WT mice. Specifically, the total distance traveled (Figure 1(e)) and average movement speed (Figure 1(f)) were significantly decreased in zQ175 mice. Additionally, immobility time was significantly increased in HD mice relative to controls (Figure 1(g)), while no significant differences were observed in freezing time between the groups (Figure 1(h)). These findings suggest that HD mice experience notable impairments in motor function, muscle strength, and general physical activity. The observed reductions in grip strength, locomotor activity, and rotarod performance suggest that both neuromuscular and coordination deficits are present at this disease stage, consistent with earlier findings.^20,21^

### Homozygous zQ175 mice exhibit reduced EDL muscle mass and impaired contractile function

**Figure 2.**
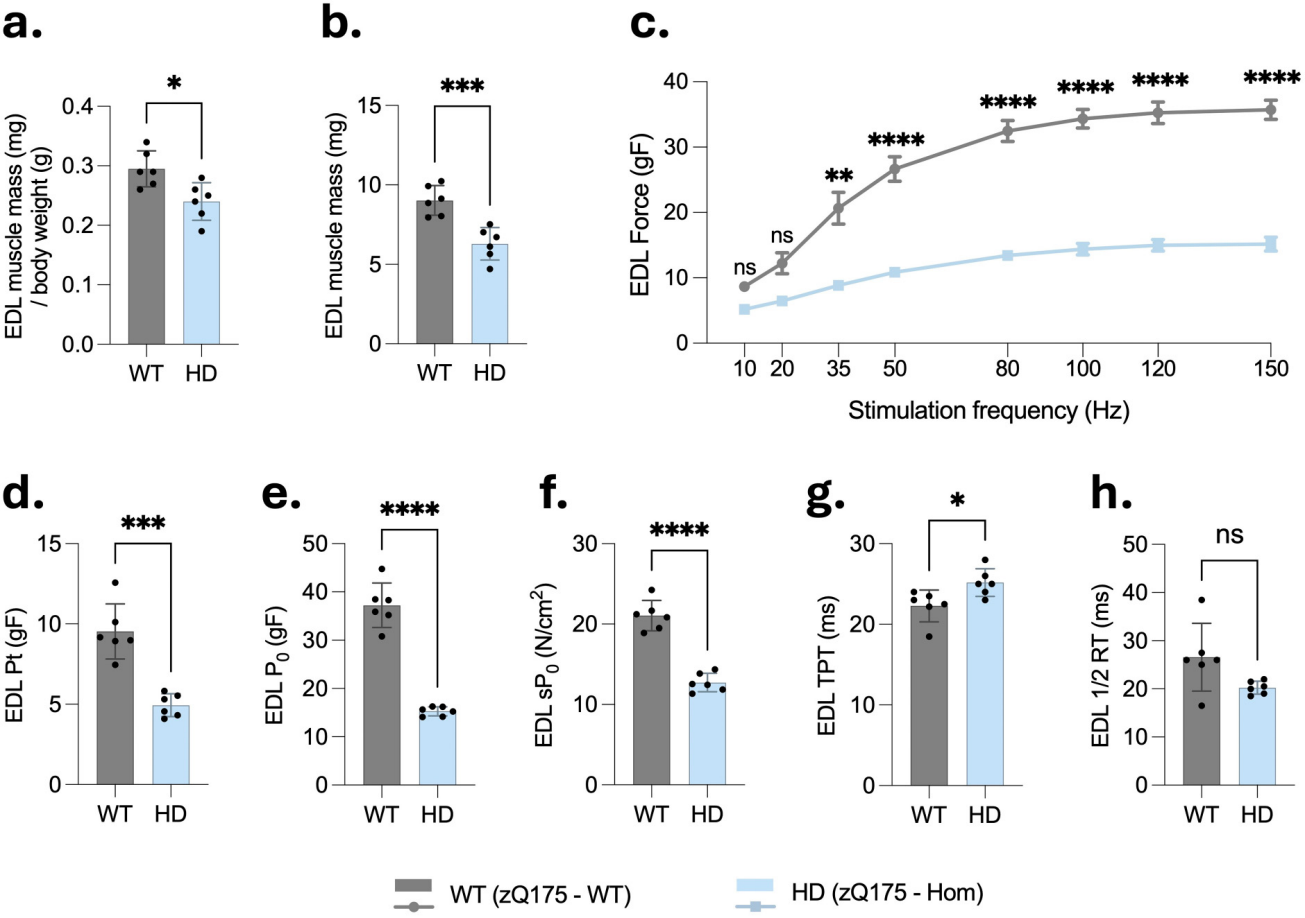
*Ex vivo* assessment reveals severe contractile dysfunction in extensor digitorum longus muscles of homozygous zQ175 mice. EDL muscles were isolated from the same male wild-type (WT) and homozygous zQ175 (HD) mice (cohort 1, n = 6 per group, all males) and subjected to *ex vivo* contractile testing. (a–b) EDL muscle mass, expressed both relative to body weight and as absolute dry mass, was significantly reduced in HD mice compared to WT controls. (c) Force-frequency curves showed a significant decrease in absolute isometric force in HD muscles across stimulation frequencies from 35 to 150 Hz. (d–e) Twitch force (Pt) and maximal tetanic force (P_0_) were significantly lower in HD mice. (f) Specific tetanic force (sP_0_; P_0_ normalized to physiological cross-sectional area) was also significantly decreased. (g) Time to peak tension (TPT; the time between stimulation onset and peak contraction) was significantly prolonged in HD mice. (h) Half-relaxation time (1/2 RT; the time taken for force to decline to 50% of its peak value) was not significantly different between genotypes. Data are presented as mean ± SD. Statistical comparisons were performed using unpaired two-tailed Student's t-test. *p < 0.05, **p < 0.01, ***p < 0.001, ****p < 0.0001; ns: not significant.

To determine whether skeletal muscle mass is reduced in HD mice, the EDL muscle was dissected, dried, and weighed from both HD and WT mice. Both the EDL muscle mass normalized to body weight (Figure 2(a)) and the absolute dry mass (Figure 2(b)) were significantly lower in zQ175 mice compared to WT controls, indicating pronounced muscle atrophy in the HD model. To further characterize muscle function, *ex vivo* contractile properties of the fast-twitch EDL muscle were assessed. This method is considered the gold standard for evaluating isolated muscle performance. The force-frequency response curves revealed that the EDL muscles from HD mice generated consistently lower force across a range of stimulation frequencies than those from WT mice (Figure 2(c)), indicating compromised muscular function. Additionally, HD mice exhibited pronounced deficits in muscle contractility. Specifically, the peak twitch force (Pt), absolute isometric force (P_0_), and specific force (sP_0_) were significantly lower in HD mice (Pt = 4.93 g, P_0_ = 15.17 g, sP_0_ = 12.84 N/cm²) compared to WT mice (Pt = 9.54 g, P_0_ = 37.91 g, sP_0_ = 21.15 N/cm²) (Figures 2(d)**–**(f)). Moreover, the time to peak tension (TPT) was significantly extended in EDL muscles from HD mice compared to WT controls (Figure 2(g)), suggesting delayed force development in HD muscle. In contrast, the half-relaxation time (½ RT) did not differ significantly between the two groups (Figure 2(h)). These findings indicate that HD mice exhibit both reduced skeletal muscle mass and impaired contractile function, consistent with muscle dysfunction observed in HD.

### Soleus muscle contractile properties are reduced in homozygous zQ175 mice

**Figure 3.**
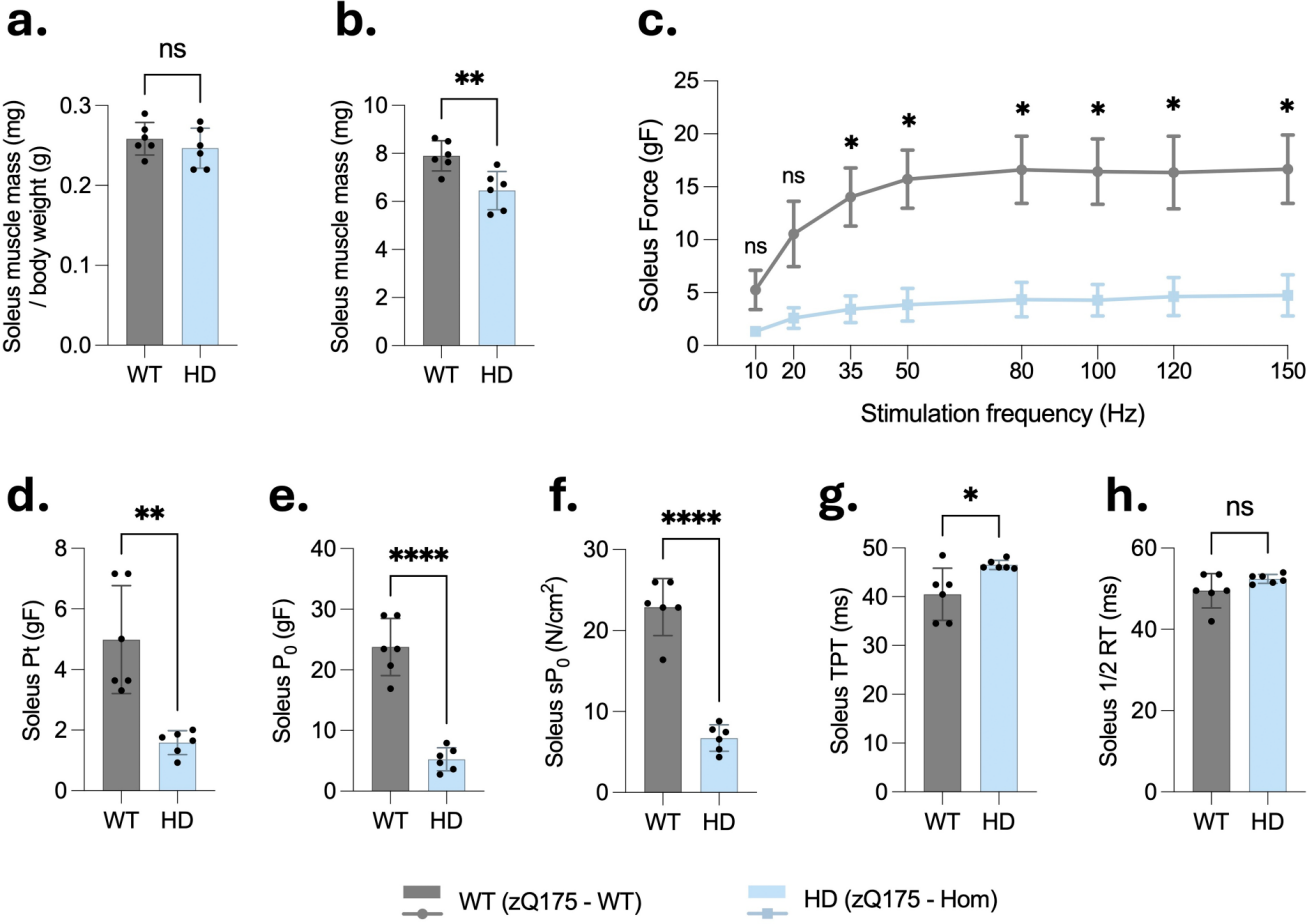
Contractile deficits in soleus muscles of 6-month-old homozygous zQ175 mice revealed by *ex vivo* functional analysis. Soleus muscles were dissected from the same 6-month-old male wild-type (WT) and homozygous zQ175 (HD) mice (cohort 1, n = 6 per group) and subjected to *ex vivo* physiological testing. (a–b) Soleus mass was recorded both relative to body weight, which did not differ significantly between groups, and as absolute dry mass, which was significantly reduced in HD mice compared to WT controls. (c) Force-frequency relationships showed significantly lower isometric force generation in HD muscles across stimulation frequencies from 35 to 150 Hz. (d–e) Peak twitch force (Pt) and maximal tetanic force (P_0_) were both significantly reduced in HD mice. (f) Specific tetanic force (sP_0_; force normalized to physiological cross-sectional area) was significantly decreased in HD mice. (g) Time to peak tension (TPT) was significantly prolonged in HD mice, whereas (h) half-relaxation time (1/2 RT) remained unchanged. Data are presented as mean ± SD. Statistical comparisons were performed using unpaired two-tailed Student's t-test. *p < 0.05, **p < 0.01, ***p < 0.001, ****p < 0.0001; ns: not significant.

The soleus muscle mass normalized to body weight did not differ significantly between HD and WT mice (Figure 3(a)). However, the absolute dry mass of the soleus was significantly reduced in HD mice compared to WT controls (Figure 3(b)). Despite the protected mass, the force-frequency curves demonstrated a significant reduction in force generation capacity in zQ175 Soleus muscles across stimulation frequencies relative to WT mice (Figure 3(c)). Furthermore, contractility was markedly impaired in HD mice. Specifically, the peak twitch force (Pt), absolute isometric force (P_0_), and specific force (sP_0_) were significantly reduced in Soleus muscles from zQ175 mice (Pt = 1.39 g, P_0_ = 4.73 g, sP_0_ = 6.07 N/cm²) compared to age-matched WT controls (Pt = 4.69 g, P_0_ = 22.57 g, sP_0_ = 22.46 N/cm²) (Figures 3(d)**–**(f)). The TPT was significantly extended in Soleus muscles from HD mice compared to WT controls (Figure 3(g)). Additionally, no significant differences were observed in the half-relaxation time (½ RT) between HD and WT mice in this muscle (Figure 3(h)), indicating that the kinetics of muscle contraction and relaxation were probably preserved despite the reduction in contractile force in Soleus muscle.

**Figure 4.**
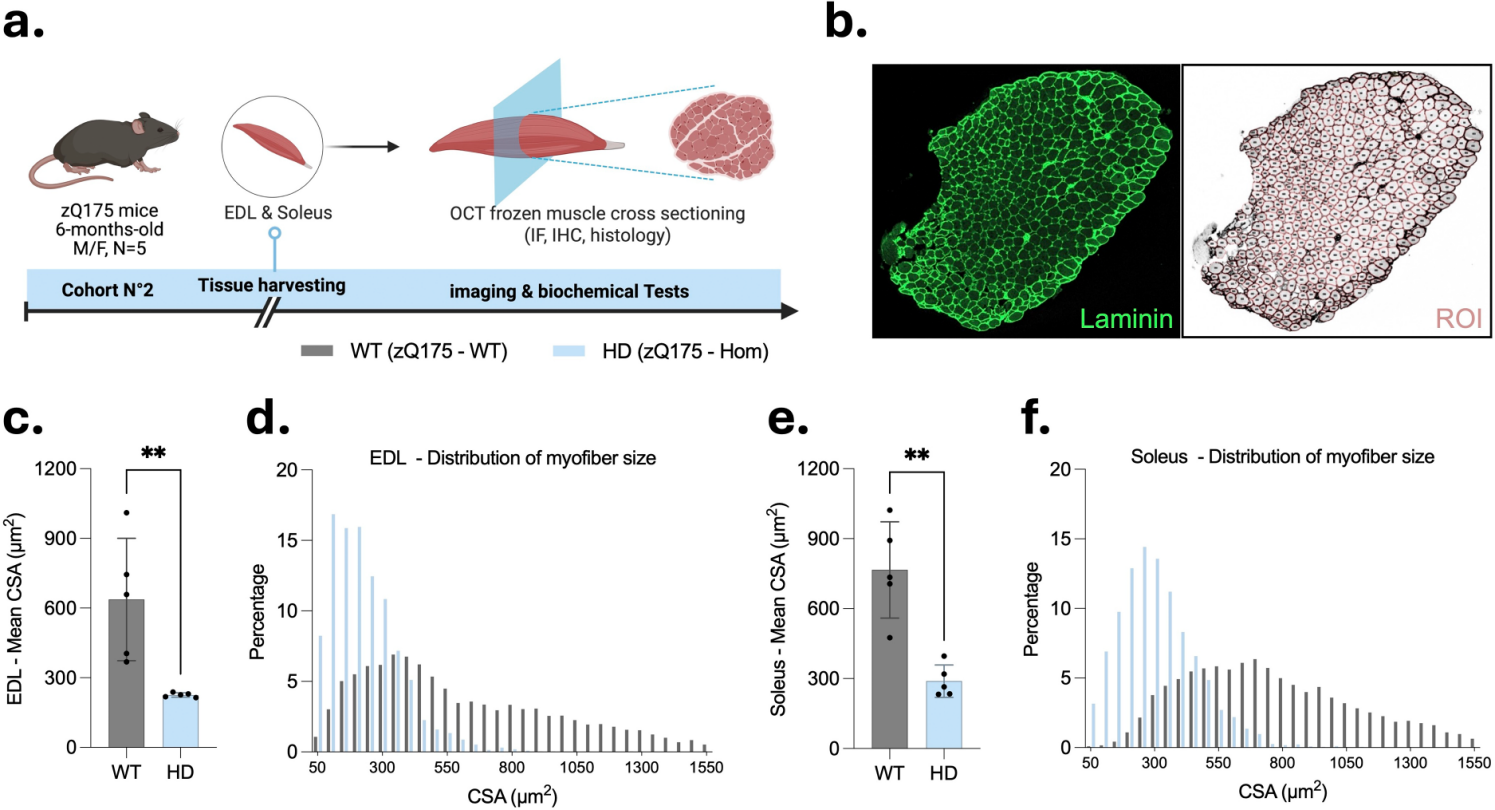
Histomorphometric analysis of skeletal muscle reveals significant myofiber atrophy in 6-month-old homozygous zQ175 mice. (a) Schematic of the experimental workflow used for histological analysis of muscle cross-sections in the second cohort, which consisted of sex and age-matched 6-month-old mice (n = 5 per genotype), with each group comprising 3 males and 2 females. No sex-related differences were observed in body weight (data not shown) or in the subsequent CSA measurements. Illustration created with BioRender.com. (b) Representative image of a laminin-stained cross-section of extensor digitorum longus (EDL) muscle from a wild-type mouse, and corresponding myofiber region-of-interest (ROI) segmentation using Cellpose and LabelToROI plugins in Fiji. (c, e) Quantification of mean myofiber cross-sectional area (CSA) in EDL (c) and Soleus (e) muscles from 6-month-old homozygous zQ175 (HD) and wild-type (WT) mice (n = 5 per group, mixed sex). Bars represent the mean ± SD, where each point corresponds to the average CSA of all myofibers measured within a single muscle cross-section from one mouse (one section per mouse was analyzed, and this average value was used as a single data point for statistical analysis to ensure independence of observations). Statistical analysis was performed using unpaired two-tailed Student's t-test (**p < 0.01). (d, f) Frequency distribution histograms showing the percentage of myofibers falling within each CSA bin for EDL (d) and Soleus (f) muscles. Histograms were generated from the same muscle cross-sections used to generate the mean CSA values in panels (c) and (e), with the CSA of all individual myofibers in each section contributing to the bin distribution (n = 5 sections per group, one per mouse).

### Histomorphometric analysis reveals severe myofiber atrophy in homozygous zQ175 mice

To evaluate skeletal muscle morphology, histological cross-sections of EDL and Soleus muscles were prepared from 6-month-old homozygous zQ175 (HD) and wild-type (WT) mice (Figure 4(a)), then processed for immunofluorescent detection of laminin to delineate myofiber boundaries (Figure 4(b)). Quantitative analysis revealed a significant reduction in myofiber CSA in HD mice compared to WT in both EDL (Figure 4(c)) and Soleus (Figure 4(e)) muscles (**p < 0.01 for both). Frequency distribution histograms (Figures 4(d) and (f)) demonstrated a leftward shift in CSA values in HD mice, indicating a greater proportion of smaller-diameter myofibers. In the EDL muscle (Figure 4(d)), the number of myofibers with a CSA < 300 μm² was approximately doubled in HD mice, while the number of larger fibers (> 550 μm²) was reduced, with complete absence of myofibers exceeding 1050 μm². Interestingly, a more marked shift was observed in the Soleus muscle (Figure 4(f)), where HD mice exhibited a ∼ 4-fold increase in small fibers (< 300 μm²) and a substantial reduction in large fibers (> 550 μm²), accompanied with no fibers above 1050 μm². Notably, the longitudinal position of the section within the muscle was not systematically standardized to the anatomical mid-belly for all specimens due to technical constraints. As myofiber diameter varies along the muscle length, with maximal CSA typically found at the mid-belly, this methodological variability may have contributed to the unexpected observation that WT soleus fibers display larger mean CSA that WT EDL fibers, which is opposite to most published reports. Such variability in sectioning, together with the limited sample size, could partly explain this discrepancy with literature values and should be taken into account when interpreting absolute CSA comparisons between muscle types, without implying any contradiction to the underlying physiology. Nevertheless, the observed differences between HD mice and their WT littermates remain robust. The genotype effect persisted after controlling for section size using ANCOVA, with no significant genotype × covariate interaction, confirming parallel regression slopes in WT and HD muscles. Furthermore, the CSA reduction in HD mice was consistent across both muscle types and evident in the entire CSA distribution, not only in mean values. Taken together, these results provide strong, quantitative evidence that myofiber atrophy is present in both fast-twitch EDL and slow-twitch Soleus muscles of 6-month-old homozygous zQ175 mice, independent of potential non-mid-belly sectioning bias.

### Cortical bone parameters were slightly altered in homozygous zQ175 mice

**Figure 5.**
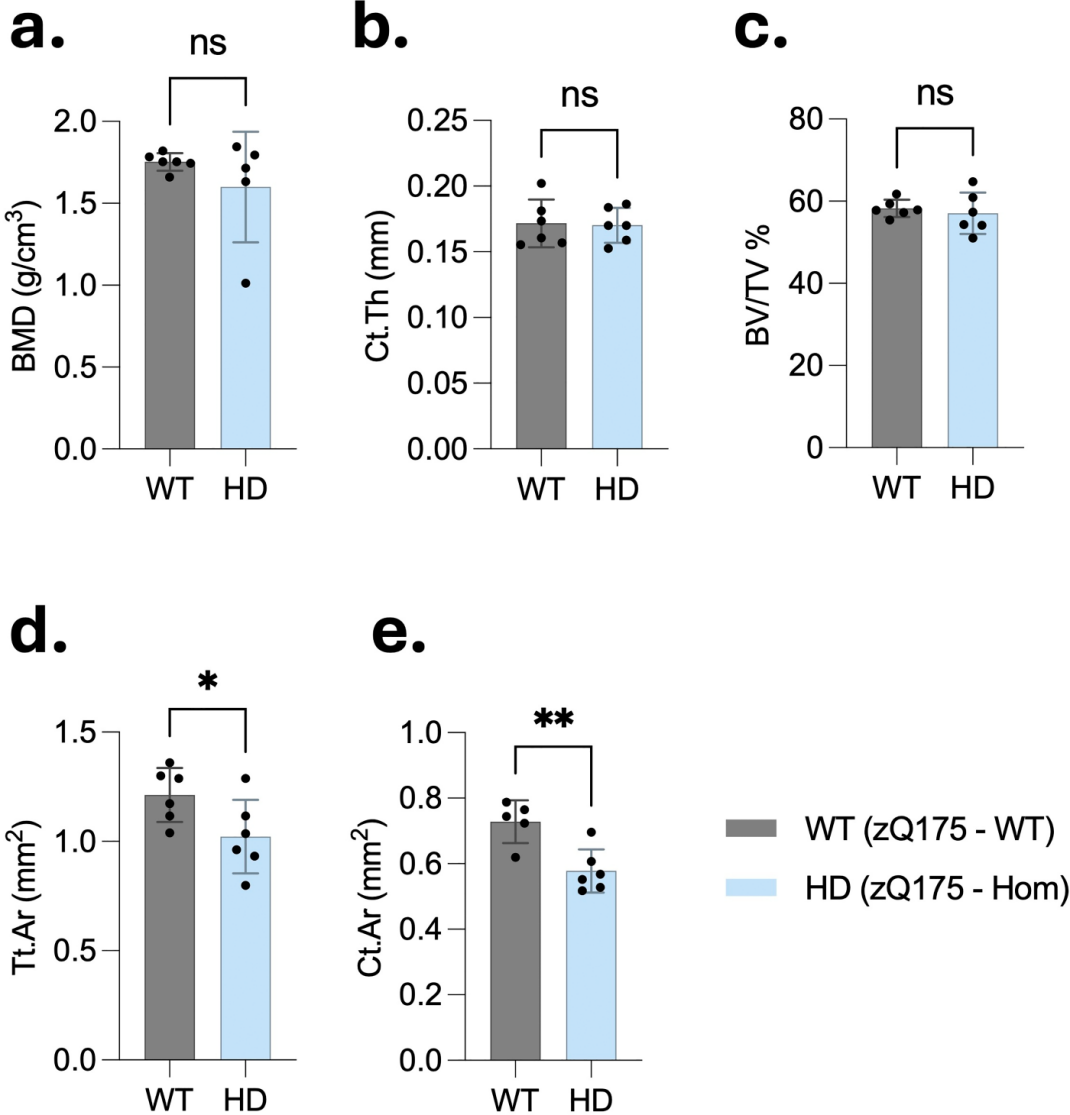
Micro-CT analysis reveals cortical bone alterations in 6-month-old homozygous zQ175 mice. Cortical bone structure was assessed in the tibia of 6-month-old wild-type (WT) and homozygous zQ175 (HD) mice using high-resolution µCT (cohort 1, n = 6 per group, all males). (a–c) No significant differences were observed in bone mineral density (BMD), cortical thickness (Ct.Th), or trabecular bone volume fraction (BV/TV: trabecular bone volume/tissue volume). (d, e) However, total cross-sectional area (Tt.Ar) and cortical bone area (Ct.Ar) were significantly reduced in HD mice compared to WT controls, indicating structural compromise of the cortical compartment. All data are presented as mean ± SD. Statistical analysis was performed using unpaired two-tailed Student's t-test. *p < 0.05, **p < 0.01; ns: not significant.

This study also investigated whether cortical BMD and microstructural parameters are altered in HD mice. Micro-computed tomography (μCT) analysis of the cortical bone revealed no significant differences in BMD between HD and WT mice (Figure 5(a)). In addition, cortical thickness (Ct.Th) and the bone volume fraction (BV/TV) remained unchanged between the groups (Figures 5(b) and (c)). However, certain structural parameters exhibited mild reductions in HD mice. Specifically, total cross-sectional area (Tt.Ar) and the cortical area (Ct.Ar) were slightly decreased in HD mice compared to WT controls (Figures 5(d) and (e)). Quantitatively, the mean Ct.Ar was 0.578 ± 0.066 mm² in HD mice versus 0.728 ± 0.111 mm² in WT mice, and the mean Tt.Ar was 1.022 ± 0.167 mm² versus 1.212 ± 0.123 mm², respectively.

**Figure 6.**
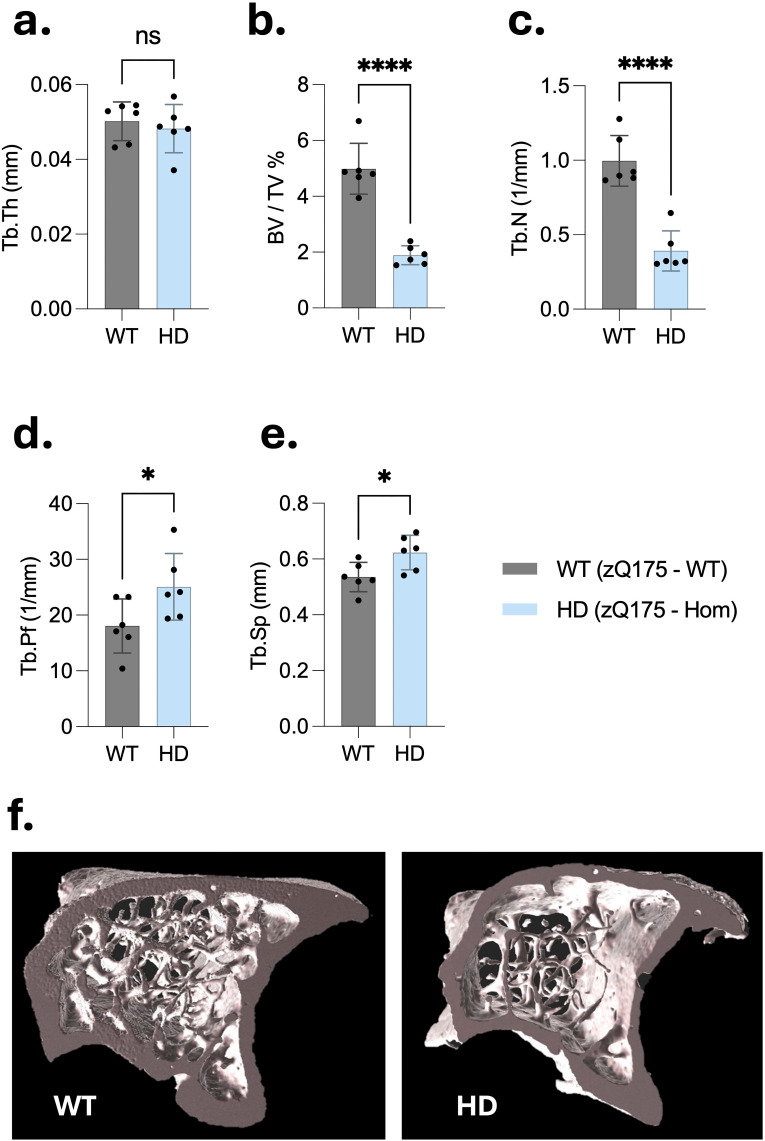
Micro-CT analysis reveals significant trabecular bone deterioration in the tibia of 6-month-old homozygous zQ175 mice. Trabecular bone microarchitecture was assessed in the proximal tibia of wild-type (WT) and homozygous zQ175 (HD) mice (cohort 1, n = 6 per group, all males) using high-resolution µCT. (a–e) plots display quantitative comparisons of key trabecular parameters between genotypes. (a) No significant difference was observed in trabecular thickness (Tb.Th). However, HD mice exhibited a significant reduction in (b) trabecular bone volume fraction (BV/TV: bone volume/tissue volume) and (c) trabecular number (Tb.N), along with (d) increased trabecular pattern factor (Tb.Pf) and (e) trabecular separation (Tb.Sp). (f) Representative 3D reconstructions of tibial trabecular bone illustrate marked loss of trabecular structure in HD mice compared to WT controls. All data are presented as mean ± SD. Statistical analysis was performed using unpaired two-tailed Student's t-test. *p < 0.05, ****p < 0.0001; ns: not significant.

### Trabecular bone parameters were substantially altered in homozygous zQ175 mice

The μCT analysis of trabecular bone architecture revealed significant alterations in HD mice compared to WT controls. Although there was no significant difference in trabecular bone thickness (Tb.Th) (Figure 6(a)), HD mice exhibited a marked reduction in trabecular bone volume fraction (BV/TV) (Figure 6(b)), and trabecular number (Tb.N) (Figure 6(c)), alongside an increase in the trabecular bone pattern factor (Tb.Pf) (Figure 6(d)), and trabecular separation (Tb.Sp) (Figure 6(e)). Quantitatively, BV/TV and Tb.N were significantly lower in HD mice (1.886 ± 0.339% and 0.391 ± 0.134 1/mm, respectively) compared to WT mice (4.987 ± 0.912% and 0.996 ± 0.170 1/mm, respectively) (Figures 6(b) and (c)). In contrast, Tb.Pf and Tb.Sp were higher in HD mice (25.062 ± 5.968 1/mm and 0.623 ± 0.0729 mm, respectively) than in WT mice (18.062 ± 4.853 1/mm and 0.535 ± 0.0666, respectively), indicating greater trabecular discontinuity in the HD mice (Figures 6(d) and (e)). Visual inspection of the μCT reconstructions revealed noticeably larger intracortical gaps in the trabecular compartment of the HD mice compared to WT controls, which is consistent with the quantitative structural data and suggestive of compromised bone integrity (Figure 6(f)). These data demonstrate that HD mice exhibit compromised trabecular bone microarchitecture, characterized by reduced bone volume and trabecular number, increased separation and structural disorganization, pointing to early-onset skeletal fragility.

## Discussion

In this study, we provide *in vivo* evidence of concurrent skeletal muscle and bone dysfunction in zQ175 mice, a widely used KI mouse model of HD. Skeletal muscle constitutes approximately 40–45% of total body mass in humans, and progressive muscle loss is a major contributor to weight loss observed in HD patients.^12^ Although previous studies have indicated that musculoskeletal abnormalities contribute significantly to HD progression,^24^ comprehensive assessments using integrated biomechanical and structural approaches have been limited in both humans and mice. To our knowledge, this is the first comprehensive study to evaluate simultaneously skeletal muscle and bone dysfunction in zQ175 mice through a combination of *ex vivo* muscle contractility testing, myofiber morphometric analysis, and μCT.

The zQ175 KI mouse model preserves the full-length human mHTT gene within the appropriate genomic context, recapitulating key features of human HD, including progressive motor deficits, body weight reduction, and behavioral impairments.^20,25,26^ Consistent with previous reports,^19,21,27–30^ we observed clear locomotor dysfunction, reduced grip strength, and impaired motor coordination in homozygous zQ175 mice, as seen also in heterozygous zQ175 mice at later ages. While some studies have suggested comparable phenotypes between heterozygous and homozygous models,^31,^^32^ others indicate that homozygous zQ175 mice exhibit more pronounced neurodegenerative and behavioral phenotypes, potentially modeling the juvenile-onset form of HD or rare mHTT homozygote carriers.^33–37^ In addition to behavioral testing, we assessed skeletal muscle functionality via *ex vivo* contractility measurements of the fast-twitch EDL and slow-twitch Soleus muscles. Both muscles displayed significant reductions in twitch force, absolute isometric force, and specific force compared to WT controls. These functional deficits were accompanied by altered force-frequency responses and prolonged TPT in EDL muscles, highlighting impaired contractile kinetics. Our findings in the zQ175 mouse model are consistent with a previous study in R6/2 and HdhQ150 mouse models, which also exhibit progressive skeletal muscle dysfunction.^18^ In those models, symptomatic mice showed impaired contractile properties in the tibialis anterior (TA) and EDL muscles, accompanied by motor unit loss in the EDL. These deficits were associated with prolonged TPT and relaxation times, suggesting a shift from fast-twitch to slow-twitch muscle fibers or impairment of Ca^2+^ handling during contraction and relaxation.^38^ The findings highlight that skeletal muscle abnormalities represent a significant component of the disease phenotype in HD.

To further investigate the structural basis of muscle dysfunction, we analyzed the myofiber cross-sectional area. While interpreting CSA data, it is important to note that the absolute mean CSA values for WT Soleus appeared larger than those for WT EDL in our dataset, which is the inverse of most published reports. This discrepancy is likely attributable to technical factors, as sectioning was not systematically standardized to a specific direction or to the anatomical mid-belly, where CSA is maximal. Because fiber size varies along the longitudinal axis with EDL mid-belly regions enriched in large type IIb fibers compared to Soleus having mostly small sized type I and IIa fibers^39,40^; sampling from non-mid-belly regions can alter absolute CSA rankings between muscle types. However, ANCOVA controlling for section size confirmed that genotype effects remained significant in both muscles and that regression slopes were parallel across WT and HD, indicating that the genotype differences reported here are robust and not driven by sectioning bias.

Indeed, the marked reduction in CSA observed in both EDL and Soleus muscles of homozygous zQ175 mice is indicative of widespread muscle atrophy in this HD model. These results align with previous findings in transgenic models such as R6/2, where muscle wasting was similarly characterized by decreased fiber diameter.^3,39–41^ However, unlike transgenic models that often display rapid and severe phenotypes due to supraphysiological mHTT transgene expression, the zQ175 mouse offers a more genetically accurate model to study HD-related muscle degeneration under endogenous regulatory control.

Our data notably reveal that muscle atrophy is observed at 6 months of age, an early-to-mid symptomatic in homozygous zQ175 mice,^26,^^42^ indicating that skeletal muscle deterioration represents an early manifestation of HD pathology rather than a late consequence of motor decline or immobility. At this age, HD mice remained active and mobile, although to a lesser extent compared to WT controls.

Physical inactivity is nevertheless a recognized contributor to musculoskeletal decline, as unloading and reduced use are sufficient to induce muscle atrophy and bone loss. Given that HD mice exhibit reduced spontaneous activity compared to WT controls (Figure 1(e)–(h)), inactivity is likely to exacerbate musculoskeletal deterioration. However, our findings and prior work suggest that inactivity alone cannot explain the phenotype. HD pathology involves intrinsic toxic effects of mHTT within muscle and bone, including transcriptional deregulation, mitochondrial dysfunction, impaired energy metabolism, dysregulated proteostasis, and neuromuscular junction (NMJ) abnormalities. Importantly, these musculoskeletal defects emerge at disease onset, a stage when locomotor hypoactivity also begins to manifest, indicating that reduced activity likely exacerbates the pathology but is not its primary driver. Instead, musculoskeletal decline reflects a parallel pathological process occurring alongside neurodegeneration, reinforcing the view that HD-associated atrophy is not merely a secondary consequence of progressive motor impairment.

The more pronounced shift toward small fiber diameters in the Soleus muscle, a predominantly oxidative and postural muscle, may reflect differential vulnerability of muscle fiber types to mHTT toxicity. This phenomenon has also been reported in other neuromuscular and mitochondrial disorders, where slow oxidative fibers, such as those predominantly found in the Soleus, show increased sensitivity to mitochondrial dysfunction, oxidative stress, and impaired calcium handling.^43–46^ These vulnerabilities may arise due to the high metabolic demands and reliance on mitochondrial respiration in type I fibers, rendering them more susceptible to energetic failure and proteostatic stress in disease conditions. The mechanistic underpinnings may involve mHTT-induced mitochondrial impairment, increased oxidative stress, and activation of proteolytic systems such as the ubiquitin-proteasome and autophagy-lysosome pathways, but also impaired neuromuscular transmission.^3,4,38,41^ Overall, these morphological alterations are consistent with the observed functional impairments and suggest that muscle atrophy, particularly affecting slow-twitch fibers, may underlie early declines in muscle performance in HD. These findings collectively validate the relevance of zQ175 mice for modeling HD-related muscle pathology and underscore myofiber CSA quantification as a sensitive and translational readout of skeletal muscle deterioration. Future investigations will be essential to determine whether interventions targeting muscle metabolism or proteostasis can mitigate fiber atrophy and enhance motor function in this model.

Beyond the weight loss,^47^ there are hidden changes in the body compartments, an obvious reduction of muscle mass, and a reduction of bone mineralization.^48,^^49^ Given the frequent co-occurrence of muscle wasting and bone loss in neuromuscular disorders,^47,48^ we also investigated cortical and trabecular bone properties using μCT. Previous clinical studies have reported reductions in BMD and increased risk of osteoporosis in HD patients^24,50^; however, the mechanisms remain poorly understood. Although overall BMD was not significantly altered in homozygous zQ175 mice, we detected reductions in Ct.Ar and total Tt.Ar, indicating compromised cortical bone structure. In the trabecular compartment, we observed significantly decreased BV/TV and Tb.N, along with increased Tb.Pf and Tb.Sp, reflecting deterioration of trabecular microarchitecture.

These findings reveal significant impairments in both muscle function and bone integrity in homozygous zQ175 mice. The combined deficits in muscle strength, fiber morphology, and bone structure underscore the systemic nature of HD pathology. This comprehensive assessment highlights the value of the zQ175 model for investigating peripheral manifestations of HD and suggests that therapeutic strategies targeting musculoskeletal tissues may offer benefit alongside approaches addressing central nervous system dysfunction.

In conclusion, homozygous zQ175 mice exhibit significant impairments in locomotor activity, grip strength, and motor coordination, accompanied by pronounced skeletal muscle and bone dysfunction. *Ex vivo* analyses revealed reduced muscle force production, and morphometric assessments indicated muscle atrophy through a shift toward smaller myofiber cross-sectional areas. Skeletal analysis by μCT further demonstrated deterioration of trabecular and cortical bone structure, with reductions in BV/TV, Tb.N, Tt.Ar, and Ct.Ar despite unchanged cortical BMD. These findings understore the systemic nature of HD pathology and highlight the value of the homozygous zQ175 model for investigating peripheral manifestations. Therapeutic strategies targeting musculoskeletal health may offer promising avenues for improving functional outcomes and quality of life in HD.
